# Evolutionary Analysis of Calcium-Dependent Protein Kinase in Five *Asteraceae* Species

**DOI:** 10.3390/plants9010032

**Published:** 2019-12-24

**Authors:** Liping Zhu, Bowen Zheng, Wangyang Song, Hongbin Li, Xiang Jin

**Affiliations:** 1 Ministry of Education Key Laboratory for Ecology of Tropical Islands, College of Life Sciences, Hainan Normal University, Haikou 571158, China; zhuliping0903@163.com (L.Z.); zhengbw0609@163.com (B.Z.); swywinner@163.com (W.S.); 2 Key Laboratory of Xinjiang Phytomedicine Resource and Utilization of Ministry of Education, College of Life Sciences, Shihezi University, Shihezi 832003, China

**Keywords:** calcium-dependent protein kinases, natural rubber producing, evolutionary analysis, *Asteraceae*, gene family expansion

## Abstract

Calcium-dependent protein kinase (CPK) is crucial in Ca^2+^ signal transduction, and is a large gene family in plants. In our previous work, we reported *Hevea brasiliensis* CPKs were important for natural rubber biosynthesis. However, this *CPK* gene family in other rubber producing plants has not been investigated. Here, we report the CPKs in five representative *Asteraceae* species, including three rubber-producing and two non-rubber species. A total of 34, 34, 40, 34 and 30 CPKs were identified from *Taraxacum koksaghyz*, *Lactuca sativa*, *Helianthus annuus*, *Chrysanthemum nankingense* and *Cynara cardunculus*, respectively. All CPKs were classified into four individual groups (group I to IV). In addition, 10 *TkCPKs*, 11 *LsCPKs*, 20 *HaCPKs*, 13 *CnCPKs* and 7 *CcCPKs* duplicated paralogs were identified. Further evolutionary analysis showed that, compared to other subfamilies, the group III had been expanded in the *Asteraceae* species, especially in the rubber-producing species. Meanwhile, the *CPKs* in group III from *Asteraceae* species tend to expand with low calcium binding capacity. This study provides a systematical evolutionary investigation of the CPKs in five representative *Asteraceae* species, suggesting that the sub-family specific expansion of CPKs might be related to natural rubber producing.

## 1. Introduction

Calcium (Ca^2+^) participates in miscellaneous signal transduction pathways as the second messenger such as stress, immune and signaling. To date, there are three major classes of Ca^2+^-binding proteins that have been characterized in higher plants, including calcium-dependent protein kinases (CPK), calmodulins (CaMs) and CaM-like proteins (CaMLs), and calcineurin B-like proteins (CBLs) [1,2]. The CPK constitutes one of the largest protein kinase families that sense the calcium signal in plants [3]. The CPKs are monomeric proteins with structures that contain four conserved domains: the *N*-terminal variable domain, serine/threonine kinase domain, auto-inhibitory junction domain and the calmodulin-like domain [4,5]. The *N*-terminal domain is highly variable and contains myristoylation or palmitoylation sites for subcellular targeting [6]. The protein kinase domain is the catalytic domain with an adenosine triphosphate (ATP) binding site, which is often followed by the auto-inhibitory domain that serves as an auto-inhibitor to switch CPKs between inactive and/or active forms depending on the level of calcium concentration [7]. Moreover, the calmodulin-like domain often contains four EF-hands for Ca^2+^ binding [8,9].

*Asteraceae* is one of the largest families in terms of the number of species and diversity of habitats colonized [10]. Nearly one in ten flowering plants are members of this *Asteraceae* family [11]. Although clearly monophyletic, there is a great deal of diversity among the members: habit varies from annual and perennial herbs to shrubs, vines, or trees; species grow in every type of habitat from lowland forests to the high alpine fell fields [12]. Moreover, *Asteraceae* species are successful colonizers of disturbed habitats and spread in extreme environments, such as deserts, salt flats and tundra [13]. This family includes many important edible, medicinal, noxious and invasive species, and genome sequencings of many *Asteraceae* species have also been reported to better excavate the potential value of *Asteraceae* [14,15,16,17,18]. However, no calcium binding protein families have been investigated in these sequenced *Asteraceae* species yet.

Natural rubber (NR) is an irreplaceable raw material used to produce a wide variety of products ranging from medical devices to aircraft tires, which rubber is mainly produced by *Hevea brasiliensis* (rubber tree), that only can be cultivated in tropical regions [19]. The increased worldwide demand of NR means that alternative, sustainable NR sources are urgently required. It was reported that more than 3000 plants could produce NR, however, most of them only contain very little rubber contents that are far from the need of industrial applications. Of these rubber-producing plants, *Taraxacum koksaghyz* (*Tks*) and *Parthenium argentatum* Gray (*Pa*), coupled with the rubber tree, are called the top three rubber-producing plants, due to their high NR contents and qualities. It is notable that *Tks* and *Pa* both belong to the *Asteraceae* family. In addition, several other *Asteraceae* species were also reported to produce NR, such as the sunflower [20]. To develop rubber-producing *Asteraceae* plants will provide excellent alternative resources of NR, benefiting from the widespread habitats and adaptability to different environments of *Asteraceae* species. Rubber-producing *Asteraceae* plants can also serve as model plants for NR biosynthesis research. Several *Tks* genes that are related to rubber production have been identified, including *cis*-prenyltransferases (cPTs) that catalyze the NR elongation [21], and small rubber particle proteins (SRPPs) that maintain the structure of the rubber particles [22]. In addition, some receptors [23] and activators [24] have been reported to be involved in rubber biosynthesis in *Asteraceae* plants. Also, root transcriptomic analysis, which focused on SNPs between low and high rubber contents *Tks* varieties, added useful information to the limited genetic data developed for *Tks* [25]. 

Our previous work had reported that protein kinases play important roles in ethylene-induced rubber producing in the rubber tree. Two kinase families (mitogen-activated protein kinase and CPK) have been investigated in the rubber tree and duplicated members which showed rubber-specific expression patterns were identified [26,27]. However, little is known about the CPK family in the rubber-producing *Asteraceae* species. Here, we performed a comprehensive evolutionary and syntenic analysis of CPKs in five sequenced *Asteraceae* plants: *Taraxacum koksaghyz*, *Lactuca sativa*, *Helianthus annuus*, *Chrysanthemum nankingense* and *Cynara cardunculus*. The phylogenetic analysis revealed that the CPKs were divided into four groups. Sequences in each group were conserved, and most of the duplicated paralogs in *Asteraceae* are under purifying selection. There are two duplicated gene pairs in *TKs*, (*TkCPK26*/*TkCPK27*; *TkCPK27*/*TkCPK31*), having accelerated evolution rates. Moreover, CPKs in group II and group III were significantly expanded in rosids and *Asteraceae* species, respectively, indicating that CPK members in these two groups might have potential species-specific functional divergency in rosids and *Asteraceae*. Meanwhile, the *CPKs* in group III of *Asteraceae* species might expand with low calcium binding capacity. Our data provide valuable information for understanding the evolution and function of CPKs in rubber-producing *Asteraceae* plants.

## 2. Results

### 2.1. Identification of CPKs in Five Asteraceae Species

Genome-wide analyses of the *Asteraceae* species allowed us to identify 34, 34, 40, 34 and 30 CPK members in *Tks*, *L. sativa*, *H. annuus*, *C. nankingense* and *C. cardunculus*. Previously reported 34 *At CPKs*, 30 *OsCPKs*, 39 *GmCPKs*, 29 *SlCPKs*, 26 *StCPKs* and 30 *HbCPKs* were obtained from corresponding databases [5,28,29,30,31,32].

Among the eleven species, the numbers of exons in CPK were not significantly different from each other in group I–III, but exon numbers in group IV were significantly higher than that in groups I–III (Table 1). Coding sequence lengths and encoded polypeptides of TkCPKs, CnCPKs, HaCPKs, LsCPKs and CcCPKs range of 1452–1779 bp/483–592 aa, 1230–1815 bp/409–604 aa, 1470–1896 bp/490–632 aa, 1464–1800 bp/488–600 aa and 1470–1824 bp/489–607 aa, respectively. In *Asteraceae* species, 22 TkCPKs, 11 CnCPKs, 25 HaCPKs, 20 LsCPKs and 18 CcCPKs contain predicted palmitoylation sites and 28 TkCPKs, 22 CnCPKs, 35 HaCPKs, 28 LsCPKs and 25 CcCPKs have putative myristoylation sites (Table 1). These CPKs may function in many physiological processes by membrane association in plants, because myristoylation or palmitoylation sites within the *N*-terminal variable domain can facilitate membrane association.

### 2.2. Phylogenetic Analysis of CPK Members

**Figure 1:**
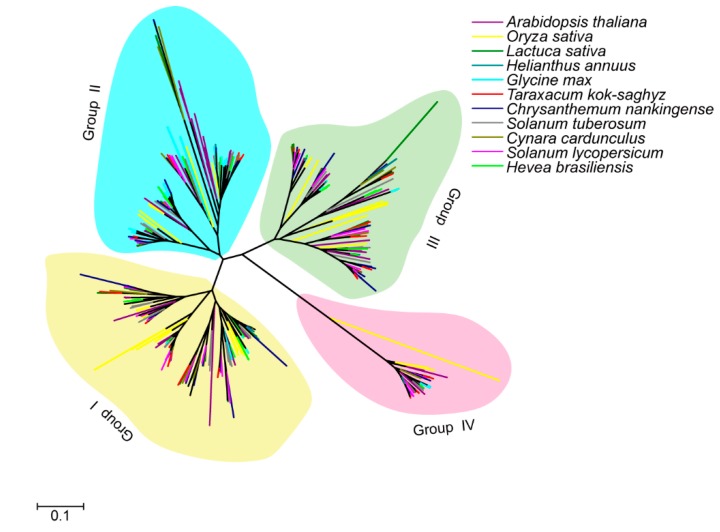
Phylogenetic tree of CPK members in eleven species. The phylogenetic tree was constructed by ClustalX 2.0 and MEGA 7.0 software using the Neighbor-Joining (NJ) method with bootstrap replicates set to 1000. The CPKs from different species are shown in different colors. The constructed phylogenetic tree showed that CPKs are clustered into four different groups: group I (yellow), group II (blue), group III (green) and group IV (pink).

The five *Asteraceae* species CPKs, rubber tree CPKs, as well as five representative model plant CPK members were used to investigate the evolutionary relationship of CPKs in *Asteraceae* plants and rubber-producing plants. The result revealed that all CPK genes fell into four different groups, group I (yellow), group II (blue), group III (green) and group IV (pink) (Figure 1). No species-specific clades and rubber-producing-specific clades were identified. However, the amounts of CPKs in different groups of eleven species are different. Group I usually has the largest number and group IV contains the fewest number of CPKs. 

### 2.3. Evolutionary Analyses of Duplicated Gene Pairs in Asteraceas Species

**Figure 2:**
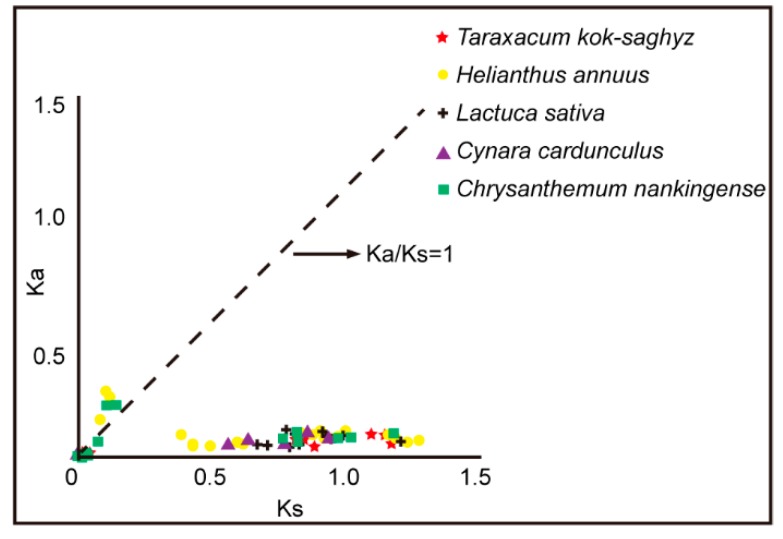
The Ka and Ks values of duplicated gene pairs in *Asteraceae*. The Ka and Ks values were calculated using Dnasp 4.0. Different colored shapes represent the Ka/Ks ratio of duplicated gene pairs from corresponding species.

To explore the evolution of the CPK family in detail, we analyzed the duplication events of the *Asteraceae* CPK gene family. A number of 10, 11, 20, 13 and 7 duplicated paralogs were identified in *Tks*, *L sativa*, *H. annuus, C. nankingense* and *C. cardunculus*, respectively. The Ka/Ks ratio was calculated to assess the selection pressure of each duplicated paralog pairs. The results showed that most duplicated *Asteraceae* CPK paralogs are under purifying selection, except six duplicated gene pairs (*HaCPK2*/*HaCPK23*, *HaCPK9*/*HaCPK19*, *HaCPK9*/*HaCPK28*, *CnCPK5*/*CnCPK28*, *CnCPK16*/*CnCPK31* and *CnCPK18*/*CnCPK26*), which are under positive selection (Figure 2, Tables S1–S5). It therefore appears that CPKs play critical roles during plant development, which requires highly conserved sequences. The paralogs under positive selection might have potential functional divergence which is involved in specific tissues, and development processes in *H. annuus* and *C. nankingense* after the emergence of *Asteraceae*.

We also addressed the question of whether these duplicated paralogs of *Asteraceae* CPKs are under an accelerated evolutionary rate. To this end, we assessed the Tajima relative rates of *Asteraceae* CPK paralogs. The *TkCPK26*/*TkCPK27* and *TkCPK27*/*TkCPK31* duplication pairs have prominently accelerate evolutionary rates (Table 2). The number of duplicated gene pairs from group III were more than that from the other three groups in the five *Asteraceae* species. Meanwhile, a total of 16 duplicated gene pairs (*LsCPK19*/*LsCPK28*, *LsCPK20*/*LsCPK31*, *LsCPK11*/*LsCPK18*, *LsCPK15*/*LsCPK29*, *LsCPK16*/*LsCPK18*, *HaCPK33*/*HaCPK34*, *HaCPK33*/*HaCPK38*, *HaCPK10*/*HaCPK16*, *HaCPK19*/*HaCPK28*, *CnCPK14*/*CnCPK25*, *CnCPK29*/*CnCPK34*, *CnCPK16*/*CnCPK31*, *CnCPK20*/*CnCPK24*, *CnCPK2*/*CnCPK30*, *CnCPK1*/*CnCPK26*, *CnCPK18*/*CnCPK26*) are under accelerated evolutionary rates in the other four *Asteraceae* species, suggesting that they potentially play specific roles. (Tables S6–S9).

### 2.4. Syntenic Analysis of CPKs from Five Asteraceae Species

**Figure 3:**
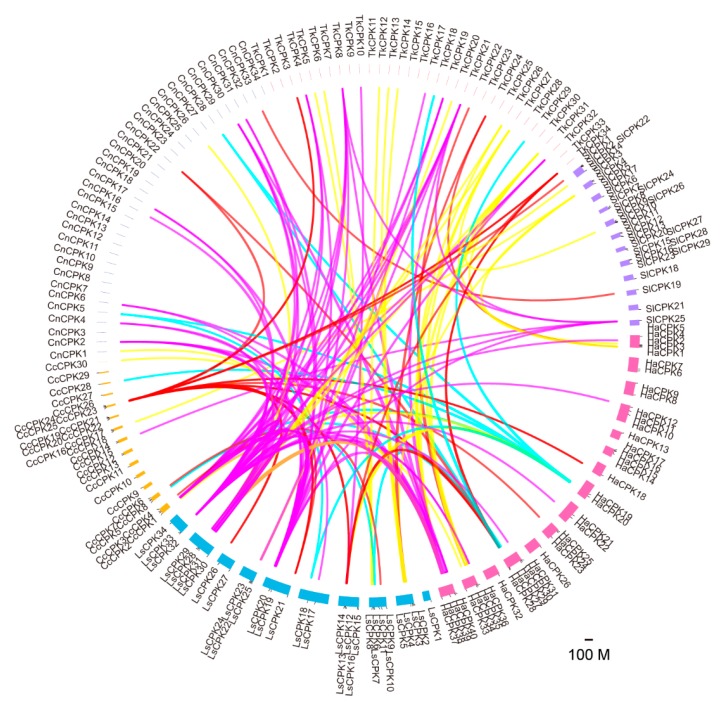
Duplication and syntenic analysis of *CPKs* in *Asteraceae* plants and *S. lycopersicum*. Chromosomes and scaffolds are shown in different colors and all *CPK* gene locations are indicated. The syntenic relationship of CPKs in four different groups are connected by different colored lines: group I (yellow), Group II (red), group III (magenta) and Group IV (blue).

The syntenic analysis of *CPK* members from *Tks*, *L sativa*, *H. annuus*, *C. nankingense*, *C. cardunculus* and *S. lycopersicum* was performed. The Circos program was used to visualize the syntenic relationship. A total of 10, 11, 20, 13 and 7 duplicated *CPK* pairs in *Tks*, *L. sativa*, *H. annuus*, *C. nankingense* and *C. cardunculus* were identified. The number of 10, 4, 7 and 1 *TkCPKs* from group I, group II, group III and group IV had syntenic relationships with *CPKs* from the other four *Asteraceae* species and tomato (Figure 3). Overall, there is a close *CPK* syntenic relationship among the five *Asteraceae* species, especially among different subgroups.

### 2.5. The CPKs in Group II and Group III Are Expanded

**Figure 4:**
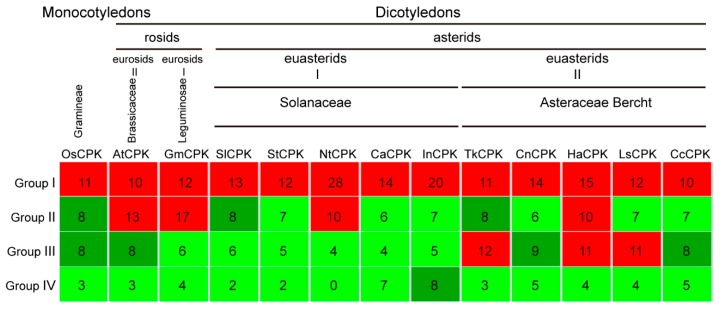
Expansion of CPKs in different groups. Thirteen species are ordered by plant classification system. The number of CPKs of four distinct groups from different species are indicated in the colored boxes. Red and green represent a large or a small number of CPK members, respectively.

To investigate the evolution of the *CPK* gene family, 39 *GmCPKs* in *Leguminosae* were chosen to represent *CPKs* in eurosids I. In addition, 34 *AtCPKs* in *Brassicaceae* represented *CPKs* in eurosids II, and 29 *Solanum lycopersicum CPKs*, 26 *Solanum tuberosum CPKs*, 28 *Nicotiana tabacum CPKs*, 31 *Capsicum annuum CPKs* and 40 *Ipomoea nil CPKs* in *Solanaceae* represented *CPKs* from euasterids I. Five *Asteraceae* species stand for *CPKs* from euasterids II and 30 *OsCPKs* in *Gramineae* represented *CPKs* in Monocotyledon. By comparing the *CPK* number in all mentioned species, we found that the *CPK* gene family has the largest number in group I and the smallest numbers in group IV. The *AtCPKs* and *GmCPKs* in rosids are significantly expanded in group II, while *CPKs* in *Asteraceae* are expanded in group III (Figure 4), indicating the potential functional divergence of expanded *CPKs* in group II and group III in rosids and *Asteraceae*, respectively. Notably, rubber-producing *Asteraceae* plants have the largest group III members (12, 11 and 11 for *Tks*, *Ha* and *Ls*), implying members of group III might be potentially involved in NR-related metabolism processes.

### 2.6. Gene Structure and Motif Distribution of CPKs

**Figure 5:**
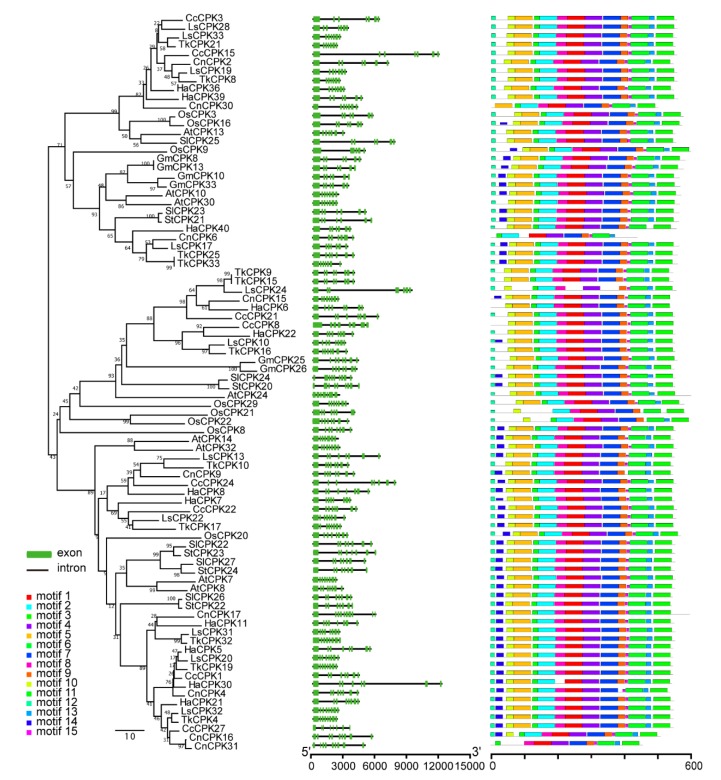
Gene structure and conserved motif distribution of group III CPKs from five model plants and five *Asteraceae* species. The phylogenetic tree-view is on the left panel. Exon–intron distribution is in the middle, in which black lines and green boxes represent introns and exons, respectively. The motif distribution is on the right panel, in which rectangles with different colors represent different conserved motifs.

Gene structure divergence plays considerable roles in gene family evolution and can be used to assess phylogenetic relationships [33,34]. To further investigate the expansion mechanism in group III, maps of exon–intron structure and motif distribution were constructed based on coding DNA sequences as well as protein sequences of group III CPKs from five model plants and five *Asteraceae* species. The result displayed a very similar exon–intron structures of ten species in group III. The first exon in most *CPK* members was the longest, followed by several shorter exons. Meanwhile, the gene structure and motif distributions showed similar patterns in other three groups (Figure 5, Figures S1–S3).

### 2.7. Motif Sequence Analysis of CPKs in Group III from Asteraceae 

**Figure 6:**
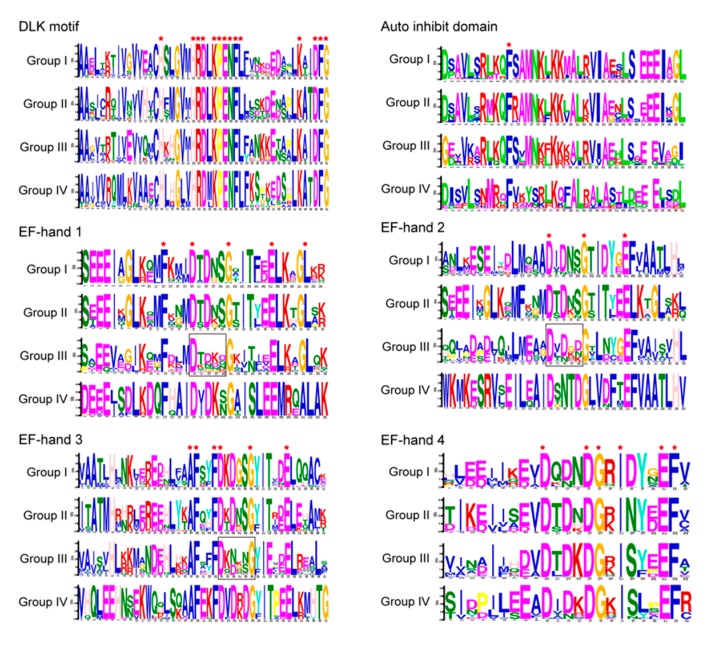
Comparison of sequence logos for conserved motifs of CPKs from the five *Asteraceae* species. Sequence logos of the consensus motifs were created using MEME online software. The height of each letter represents the frequency of amino acids at corresponding position. The red star means all CPK members have the exact same amino acid in corresponding site. Black frames showed the amino acid sites with much lower conservation in EF-hand 1, 2 and 3 of group III.

The *CPK* members of *Asteraceae* were significantly expanded in group III. The exon–intron structure and motif distribution analysis showed that all *CPKs* in group III from *Asteraceae* were conservative (Figure 5). To further explore the tendency of the CPKs’ expansion in group III, the detailed information of conserved motifs (amino acid sequences) were analyzed (Figure 6). The result showed that the DLK motif and auto-inhibitory domain of all four groups were highly conserved. However, lower conservativeness was observed in EF-hand 1 to EF-hand 3 of group III CPKs from five *Asteraceae* species. In *Asteraceae*, the “D1-X-D3-X-S5” regions of group III in EF-hand 1 to EF-hand 3 have an obviously lower convergence than that of group I, II and IV. (framed in Figure 6). The Ca^2+^-binding sites of the EF-hands were reported to be D1-D3-S5-E12 (EF-hand 1), D1-D3-S5-E12 (EF-hand 2), D1-D3-S5-E12 (EF-hand 3), and D1-D3-D5-E12 (EF-hand 4) [35]. 

It seems that group III CPKs from *Asteraceae* might have lower calcium binding capacity than other groups since the amino acids of the EF loop region participating in Ca^2+^-binding.

## 3. Discussion

### 3.1. Identification and Characteristics of CPKs in Asteraceae Species

Genome-wide identification of the CPK family has been conducted in various higher plants [28,29,30,31,32,36,37,38,39]. A total of 34, 34, 40, 34 and 30 novel CPKs and 10, 11, 20, 13 and 7 duplication gene pairs in *Tks*, *L sativa*, *H. annuus, C. nankingense* and *C. cardunculus* were identified. (Table 2, Tables S1–S5). Four species have a similar number of CPKs to that of *Arabidopsis* and rice, expect *H. annuus*, in which the CPK number significantly expanded, which may be ascribed to a much more complex evolutionary history experienced by sunflower with a lineage-specific whole-genome duplication (WGD) event around 29 million years ago [14]. In the five *Asteraceae* species studied in this study, an additional WGD event had been identified only in *H. annuus*, but not in the other four species [14,15,16,17,18].

Gene structure analysis of *CPKs* showed that the first exon in most *CPKs* was the longest one, followed by several shorter exons. The exon number in the four sub-groups were different, *CPKs* in group IV had more but shorter exons than groups I–III. The exon–intron patterns were similar between *CPKs* belonging to the same evolutionary groups (Table 1, Figure 5). Further, duplicated *CPK* gene pairs had highly conserved exon–intron patterns, which may also impact on the functional similarities and/or redundancy between these duplicated genes.

The CPK sequences among all higher plants are highly conserved, particularly in the protein kinase domain, the auto-inhibitory domain and the four EF-hand domains [40]. The CPKs of five *Asteraceae* species in this study are also highly conserved. Gene structure and motif distribution analyses of CPKs showed that the members in the same group shared similar distribution patterns of exon–introns and motifs (Figure 5 and Figures S1–S3).

Moreover, the Ka/Ks ratio among paralogs in five *Asteraceae* species demonstrated that evolutionary pressure for these sequences was maintained as most Ka/Ks ratios are less than 1 (Figure 2), indicating that these CPKs are under purifying selection.

### 3.2. Phylogenetic Analysis and Group-Specific Expansion of CPKs in Asteraceae Species

The CPKs in group IV appeared to diverge from the common ancestor with algae; group III formed a clade separate from groups I and II, while the split between group I and II appeared to be the most recent evolutionary event. The phylogenetic tree of CPK members in eleven species revealed that the group IV have the longest main branch followed by group III, and the branch of group II is the shortest (Figure 1). Furthermore, the exon–intron numbers and distribution in group IV were also different from the other three groups (Table 1, Figure 5 and Figures S1–S3), supporting the hypothesis that group IV CPKs form a separate clade of earlier lineage [32].

The CPK gene family has expanded greatly from four genes in the land plant ancestor, and less than 11 genes in green algae to approximately 30–40 members among angiosperms. Our phylogenetic analysis provides insights regarding the evolutionary relationship and group-specific expansion of CPKs from *Asteraceae* and rosids. CPKs in group II and group III are significantly expanded in rosids and *Asteraceae*, respectively (Figure 4). Gene replication contributes to the expansion of gene families. In these five *Asteraceae* species, the numbers of paralogs in group III were much higher than in other groups, indicating that gene replication was the main reason of the group III CPKs’ expansion.

The *AtCPK10* and *AtCPK30* play a central role in regulating primary nitrate responses and controlling of primary transcription by the RNA sequencing [41], suggesting that *TkCPK25*/*TkCPK33*, located in the same phylogenetic tree branch with *AtCPK10* and *AtCPK30*, might also take part in the primary transcription regulation. In addition, *TkCPK4*/*TkCPK19* and *TkCPK4*/*TkCPK32* may participate in drought stress regulation since the ortholog gene *AtCPK8* functions in ABA-mediated stomatal movement in response to drought stress through the regulation of catalase 3 [42]. *AtCPK24* could negatively regulate pollen tube growth by inhibiting K^+^ inward currents [43], indicating that *TkCPK9*, *TkCPK15* and *TkCPK16* might also be involved in the development of pollen tube. Notably, within the five *Asteraceae* species, more group III members were observed in the three rubber-producing plants (12 for *Tks*, 11 for *H. annuus* and 11 for *L. sativa*), compared to non-rubber species (Figure 4). For rubber-producing plants, the *HbCPKs* also show a slightly expansion in group III (nine members) compared with other groups [32], indicating that there might be some potential roles for group III CPKs in NR biosynthesis. 

Previous research investigated the sequence degeneration of group III CPK Ca^2+^-binding sites, showing that five AtCPKs (CPK 7, 8, 10, 13 and 32) have lower or no calcium sensitivity [44]. All these weak CPKs carry one or two altered EF-hand motif(s), suggesting that the degeneration of the EF-hand motifs can greatly influence the calcium loading. *AtCPK13*, a member of group III, inhibits stomatal opening under light-induced conditions [45], indicating their orthologs *TkCPK8*/*TkCPK21* may also be involved in a similar pathway. The expanded group III CPKs from *Asteraceae* exhibited less conservative in EF-hands of “D-X-D-X-S” region than other three groups (framed in Figure 6). Unlike EF-hands 1 to 3, EF-hands 4 of *Asteraceae* group III CPKs still showed high conservation, implying their importance to the CPK Ca^2+^-binding capacity.

## 4. Materials and Methods

### 4.1. Identification and Characteristics of CPK Members in Five Representative Asteraceae Species

The protein sequences of CPKs from *Arabidopsis* and rice served as a query sequence to perform the local BLASTP program for identifying CPK members in *Tks*, *L. sativa*, *H. annuus*, *C. nankingense* and *C. cardunculus* (e-value < 1 × 10^−5^).

InterProScan (http://www.ebi.ac.uk/interpro/) was used to determine whether all putative CPK candidates contain the protein kinase domain (PF00069) and EF-hand_7 domain (PF13499). CPK members from five representative model species and the rubber tree are derived from previous studies [5,28,29,30,31,32].

The myristoylation site and palmitoylation site were predicted by software TermiNator (https://bioweb.i2bc.paris-saclay.fr/terminator3/).

### 4.2. Phylogenetic Tree Construction

Amino acid sequences of CPKs from *A. thaliana*, *O. sativa*, *L. sativa*, *H. annuus*, *G. max*, *T. koksaghyz*, *C. nankingense*, *S. tuberosum*, *S. lycopersicum*, *C. cardunculus* and *H. brasiliensis* were aligned by ClustalX 2.0. The phylogenetic tree was constructed using MEGA 7.0 using Neighbor-Joining method, 1000 bootstrap replications [46].

### 4.3. Duplication Event and Syntenic Analysis

Paralogs of CPKs from five *Asteraceae* species were determined by multiple sequence alignment with the amino acid identification > 80%. The Ka/Ks ratios for these *CPK* paralogs were calculated to evaluate the selection pressure; the ratio > 1, < 1, or = 1 indicates positive, negative or neutral evolution, respectively. The Ka/Ks ratios of these paralogs were calculated using Dnasp 4.0 software [47]. Tajima relative rate tests were detected by MEGA 7.0 using the amino acid sequences of the duplicated CPK pairs [48]. The result of local BLASTp program (with an E-value setting of 1 × 10^−10^) and the sorted GFF profiles (with four columns, the first column is chromosome name, the second column is gene name, the third column is gene starting position and the last column is gene ending position) were then submitted to the MCScan program to identify the syntenic relationships of paralogs and/or orthologs of CPKs among six species [49]. Circos program [50] was used for visualizing the syntenic results.

### 4.4. Gene Structure and Motif Distribution Analysis

The gene structures of CPK members from ten species were constructed using TBtools JRE1.6 [51] based on the genomic sequence and coding DNA sequences corresponding to each predicted gene. The conserved motifs for all CPK protein sequences and conserved CPK sequence motif logos of five *Asteraceae* species were detected by Multiple Expectation Maximization for Motif Elicitation (MEME) online tools (http://meme.sdsc.edu/meme/intro.html).

## 5. Conclusions

In summary, our study provides a comprehensive evolutionary and systematical analysis of CPK members in five representative *Asteraceae* species, showing that for the representative rubber-producing *Asteraceae* plant Tks, duplicated gene pairs were under purifying selection pressure and two TkCPK duplication paralogs had an accelerated evolutionary rate. By comparing the CPK numbers in four groups, we found that CPKs in group II and group III were significantly expanded in rosids and rubber-producing *Asteraceae* plants, indicating that potential functional divergence of expanded CPKs in group II and group III in rosids and rubber-producing *Asteraceae* plants, respectively. Further gene structure and motif distribution analyses in group III revealed that the exon–intron and motif distribution were similar and conserved. Detailed conserved motif logos analysis revealed that CPKs in group III of *Asteraceae* species have lower amino acid conservation in EF-hand I to III, indicating that they might have lower calcium binding ability than the other three groups. Our data provide a systematical evolutionary investigation of the CPKs in five representative *Asteraceae* species, suggesting sub-family specific expansion of CPKs might be related to natural rubber producing. 

## Figures and Tables

**Table 1:** Detailed information of calcium-dependent protein kinases (CPKs) in this study.

CPKs ^a^	Species	Exon Number				CDS	aa	Pal ^b^	Myr ^b^
		I	II	III	IV				
*OsCPKs*	*Oryza sativa*	3–8	6–8	5–8	11–12	1539–1839	513–613	14	22
*AtCPKs*	*Arabidopsis thaliana*	6–7	7–9	7–8	12–13	1455–1941	484–646	21	28
*GmCPKs*	*Glycine max*	7	5–8	7–8	10–13	1377–1788	459–596	21	28
*SlCPKs*	*Solanum lycopersicum*	7–8	5–9	7–9	12	1290–1797	430–599	15	23
*StCPKs*	*Solanum tuberosum*	7–8	8–9	7–9	12	1530–2626	510–638	13	22
*TkCPKs*	*Taraxacum koksaghyz*	6–8	8	7–8	12	145–1779	483–592	22	28
*CnCPKs*	*Chrysanthemum nankingense*	6–9	8–9	7–10	8–12	1230–1815	409–604	11	22
*HaCPKs*	*Helianthus annuus*	7–9	7–8	7–8	8–12	1470–1896	490–632	25	35
*LsCPKs*	*Lactuca sativa*	6–7	7–8	7–8	8–12	1464–1800	488–600	20	28
*CcCPKs*	*Cynara cardunculus*	7	7–8	7–8	8–12	1470–1824	489–607	18	25
*HbCPKs*	*Hevea brasiliensis*	7	8–9	7–10	12	1110–1773	369–590	18	25

**^a^** All sequences used in this work are provided in Appendix A. **^b^** Pal: palmitoylation; Myr: myristoylation; The palmitoylation and myristoylation sites were predicted using TermiNator (https://bioweb.i2bc.paris-saclay.fr/terminator3/).

**Table 2:** Tajima relative rate tests of CPK gene pairs in *Taraxacum koksaghyza*.

Testing Group ^a^	Group	Mt ^b^	M1 ^c^	M2 ^d^	χ^2^	*p* ^e^
TkCPK7/TkCPK11 with SlCPK20	II	408	0	0	0.00	1.00000
TkCPK9/TkCPK15 with SlCPK24	III	357	0	0	0.00	1.00000
TkCPK25/TkCPK33 with SlCPK23	III	452	0	1	1.00	0.31731
TkCPK26/TkCPK31 with SlCPK8	I	445	0	0	0.00	1.00000
TkCPK8/TkCPK21 with SlCPK25	III	454	13	13	0.00	1.00000
TkCPK26/TkCPK27 with SlCPK8	I	417	8	28	11.11	0.00086
TkCPK27/TkCPK31 with SlCPK8	I	417	8	28	11.11	0.00086
TkCPK4/TkCPK19 with SlCPK26	III	425	16	11	0.93	0.33592
TkCPK4/TkCPK32 with SlCPK26	III	409	16	27	2.81	0.09345
TkCPK3/TkCPK28 with SlCPK18	II	380	21	20	0.02	0.87590

^a^ The Tajima relative rate test was used to examine the equality of evolutionary rate between *Tks* paralogs; ^b^ Mt is the sum of the identical sites in all three sequences tested; ^c^ M1 is the number of unique differences in the first paralog; ^d^ M2 is the number of unique differences in the second paralog; ^e^ If *p* < 0.05, the test rejects the equal substitution rates between the two duplicates and infers that one of the two duplicates has an accelerated evolutionary rate.

## Supplementary Materials

The following are available online at https://www.mdpi.com/2223-7747/9/1/32/s1, Figure S1: Gene structure and conserved motif distribution of CPKs from group I., Figure S2: Gene structure and conserved motif distribution of CPKs from group II., Figure S3: Gene structure and conserved motif distribution of CPKs from group IV., Table S1: The Ka/Ks ratios for duplicated *CPK* genes in *T. koksaghyz*, Table S2: The Ka/Ks ratios for duplicated *CPK* genes in *H. annuus*, Table S3: The Ka/Ks ratios for duplicated *CPK* genes in *L. sativa*, Table S4: The Ka/Ks ratios for duplicated *CPK* genes in *C. cardunculus*, Table S5: The Ka/Ks ratios for duplicated *CPK* genes in *C. nankingense*, Table S6: Tajima relative rate tests of *CPK* gene pairs in *H. annuus*, Table S7: Tajima relative rate tests of *CPK* gene pairs in *L. sativa*, Table S8: Tajima relative rate tests of *CPK* gene pairs in *C. cardunculus*, Table S9: Tajima relative rate tests of *CPK* gene pairs in *C. nankingense*.

## Abbreviations

CPK	calcium-dependent protein kinases
CaM	calmodulins
CaML	caM-like proteins
CBL	calcineurin B-like proteins
NR	natural rubber
cPTs	*cis*-prenyltransferases
SRPPs	small rubber particle proteins
SNP	single nucleotide polymorphism
Pal	palmitoylation
Myr	myristoylation
CDS	coding sequence
Ka	nonsynonymous substitution rate
Ks	synonymous substitutions rate
NJ	Neighbour-Joining
MEME	Multiple Expectation Maximization for Motif Elicitation
WGD	whole-genome duplication

## Appendix A

Appendix A provides all CPK sequences used in this study.
