# Supplementary material for: Evolutionary Analysis of Calcium-Dependent Protein Kinase in Five Asteraceae Species
Source: Plants (Basel). 2019 Dec 24;9(1):32. doi: 10.3390/plants9010032 (PMC7020201; doi:10.3390/plants9010032)
Supplement: Supplementary file 1 [file plants-09-00032-s001.zip › Supplementary Files_20191216.docx]

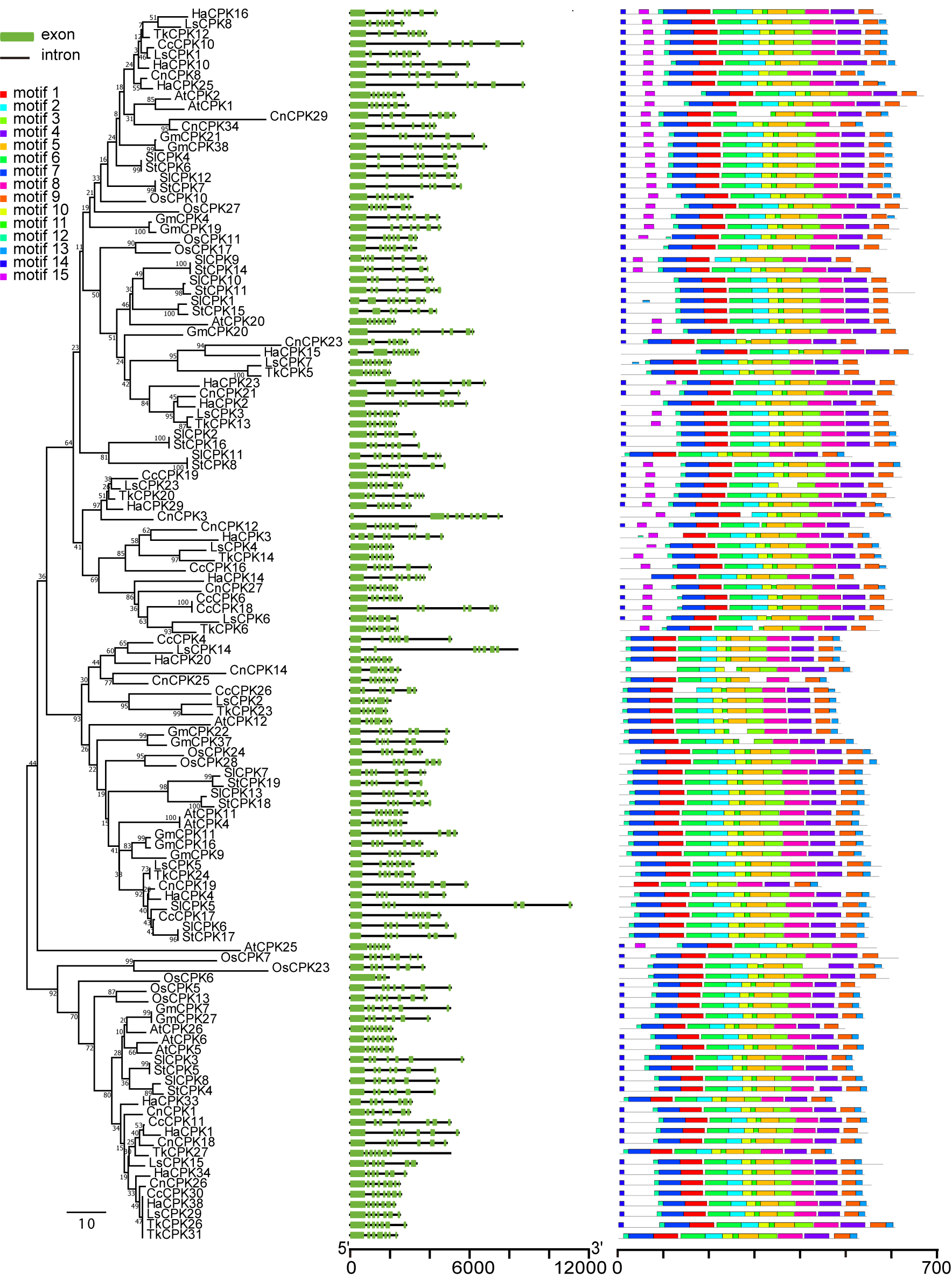


**Figure S1.** Gene structure and conserved motif distribution from group I. Tree-view is on the left panel. Exon-intron distribution is in the middle, in which the black lines and green boxes represent introns and exons, respectively. The motif distribution is on the right side, in which rectangles with different colours represent different conserved motifs.


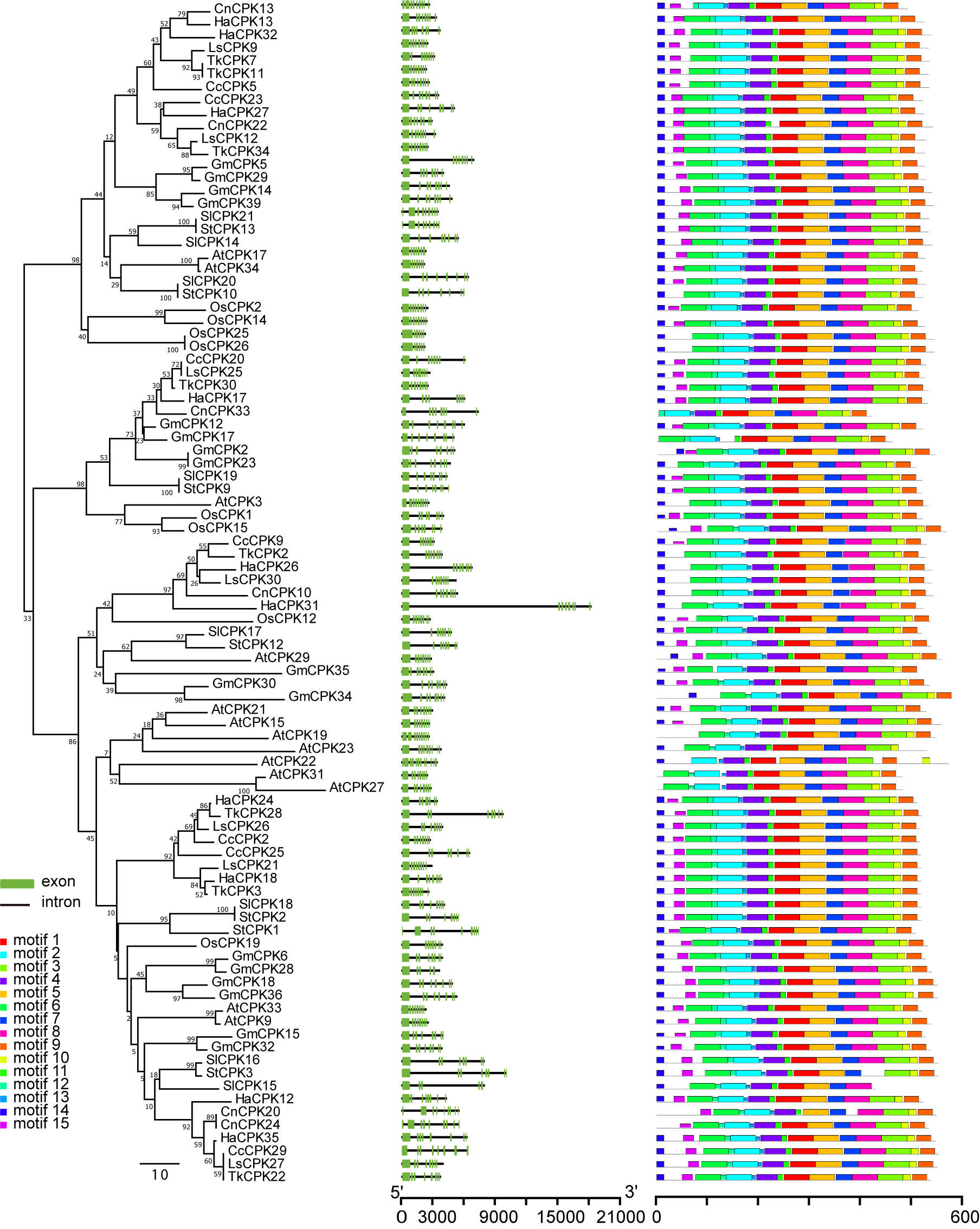


**Figure S2.** Gene structure and conserved motif distribution of CPKs from group II. Tree-view is on the left panel. Exon-intron distribution is in the middle, in which the black lines and green boxes represent introns and exons, respectively. The motif distribution is on the right side, in which rectangles with different colours represent different conserved motifs.


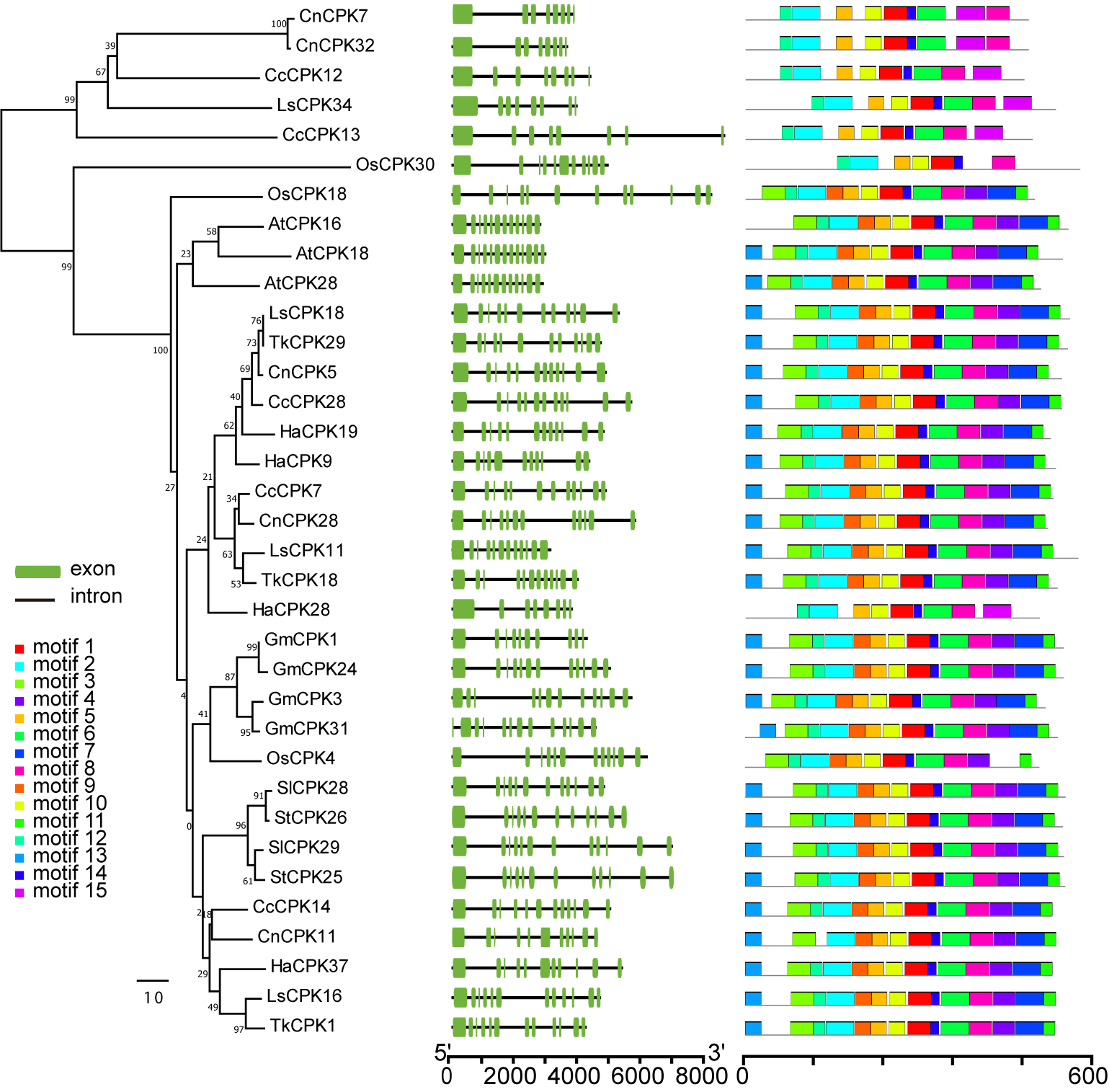


**Figure S3.** Gene structure and conserved motif distribution of CPKs from group IV. Tree-view is on the left panel. Exon-intron distribution is in the middle, in which the black lines and green boxes represent introns and exons, respectively. The motif distribution is on the right side, in which rectangles with different colours represent different conserved motifs.

**Table S1.** The Ka/Ks ratios for duplicated *CPK* genes in *T. kok-saghyz*.

| Paralogues | Ka | Ks | Ka/Ks | Selective Pressure |
| --- | --- | --- | --- | --- |
| *TkCPK3-TkCPK28* | 0.0828 | 1.0970 | 0.0755 | Purifying selection |
| *TkCPK4-TkCPK19* | 0.0473 | 1.1755 | 0.0402 | Purifying selection |
| *TkCPK4-TkCPK32* | 0.0767 | 1.1620 | 0.0660 | Purifying selection |
| *TkCPK7-TkCPK11* | 0.0000 | 0.0402 | 0.0000 | Purifying selection |
| *TkCPK8-TkCPK21* | 0.0374 | 0.8780 | 0.0426 | Purifying selection |
| *TkCPK9-TkCPK15* | 0.0008 | 0.0118 | 0.0678 | Purifying selection |
| *TkCPK25-TkCPK33* | 0.0008 | 0.0164 | 0.0488 | Purifying selection |
| *TkCPK26-TkCPK27* | 0.0579 | 0.8116 | 0.0713 | Purifying selection |
| *TkCPK26-TkCPK31* | 0.0000 | 0.0061 | 0.0000 | Purifying selection |
| *TkCPK27-TkCPK31* | 0.0577 | 0.8302 | 0.0695 | Purifying selection |

Ka: non-synonymous substitution rate; Ks: synonymous substitution rate.

**Table S2.** The Ka/Ks ratios for duplicated *CPK* genes in *H. annuus*.

| **Paralogues** | **Ka** | **Ks** | **Ka/Ks** | **Selective Pressure** |
| --- | --- | --- | --- | --- |
| *HaCPK1-HaCPK33* | 0.0644 | 1.2129 | 0.0531 | Purifying selection |
| *HaCPK2-HaCPK23* | 0.2670 | 0.1235 | 2.1619 | Positive selection |
| *HaCPK5-HaCPK11* | 0.0801 | 0.9009 | 0.0889 | Purifying selection |
| *HaCPK5-HaCPK21* | 0.0473 | 1.2575 | 0.0376 | Purifying selection |
| *HaCPK5-HaCPK30* | 0.0305 | 0.5139 | 0.0594 | Purifying selection |
| *HaCPK9-HaCPK19* | 0.1467 | 0.0983 | 1.4924 | Positive selection |
| *HaCPK9-HaCPK28* | 0.2405 | 0.1343 | 1.7908 | Positive selection |
| *HaCPK10-HaCPK16* | 0.0935 | 0.9307 | 0.1005 | Purifying selection |
| *HaCPK10-HaCPK25* | 0.0346 | 0.4517 | 0.0766 | Purifying selection |
| *HaCPK11-HaCPK21* | 0.0825 | 1.1843 | 0.0697 | Purifying selection |
| *HaCPK11-HaCPK30* | 0.0879 | 0.8543 | 0.1029 | Purifying selection |
| *HaCPK12-HaCPK35* | 0.0468 | 0.6130 | 0.0763 | Purifying selection |
| *HaCPK13-HaCPK32* | 0.0360 | 0.4466 | 0.0806 | Purifying selection |
| *HaCPK18-HaCPK24* | 0.0696 | 0.9930 | 0.0701 | Purifying selection |
| *HaCPK19-HaCPK28* | 0.0975 | 1.0218 | 0.0954 | Purifying selection |
| *HaCPK21-HaCPK30* | 0.0610 | 0.9672 | 0.0631 | Purifying selection |
| *HaCPK26-HaCPK31* | 0.0803 | 0.4088 | 0.1964 | Purifying selection |
| *HaCPK33-HaCPK34* | 0.0376 | 0.6378 | 0.0590 | Purifying selection |
| *HaCPK33-HaCPK38* | 0.0559 | 1.2960 | 0.0431 | Purifying selection |
| *HaCPK36-HaCPK39* | 0.0610 | 0.9410 | 0.0648 | Purifying selection |

Ka: non-synonymous substitution rate; Ks: synonymous substitution rate.

**Table S3.** The Ka/Ks ratios for duplicated *CPK* genes in *L. sativa*.

| **Paralogues** | **Ka** | **Ks** | **Ka/Ks** | **Selective Pressure** |
| --- | --- | --- | --- | --- |
| *LsCPK1-LsCPK8* | 0.0599 | 0.8414 | 0.0712 | Purifying selection |
| *LsCPK11-LsCPK18* | 0.1103 | 0.7966 | 0.1385 | Purifying selection |
| *LsCPK15-LsCPK29* | 0.0883 | 0.8229 | 0.1073 | Purifying selection |
| *LsCPK16-LsCPK18* | 0.0975 | 0.9348 | 0.1043 | Purifying selection |
| *LsCPK19-LsCPK28* | 0.0465 | 0.7171 | 0.0648 | Purifying selection |
| *LsCPK19-LsCPK33* | 0.0437 | 0.8028 | 0.0544 | Purifying selection |
| *LsCPK20-LsCPK31* | 0.0762 | 0.9648 | 0.0790 | Purifying selection |
| *LsCPK20-LsCPK32* | 0.0522 | 1.2216 | 0.0427 | Purifying selection |
| *LsCPK21-LsCPK26* | 0.0723 | 0.9615 | 0.0752 | Purifying selection |
| *LsCPK28-LsCPK33* | 0.0469 | 0.6817 | 0.0688 | Purifying selection |
| *LsCPK31-LsCPK32* | 0.0682 | 1.0057 | 0.0678 | Purifying selection |

Ka: non-synonymous substitution rate; Ks: synonymous substitution rate.

**Table S4.** The Ka/Ks ratios for duplicated *CPK* genes in *C. cardunculus*.

| **Paralogues** | **Ka** | **Ks** | **Ka/Ks** | **Selective Pressure** |
| --- | --- | --- | --- | --- |
| *CcCPK1-CcCPK27* | 0.0408 | 0.7831 | 0.0521 | Purifying selection |
| *CcCPK2-CcCPK25* | 0.0731 | 0.9493 | 0.0770 | Purifying selection |
| *CcCPK3-CcCPK15* | 0.0389 | 0.5690 | 0.0684 | Purifying selection |
| *CcCPK5-CcCPK23* | 0.0982 | 0.8700 | 0.1129 | Purifying selection |
| *CcCPK6-CcCPK18* | 0.0000 | 0.0000 | 1.0000 | Neutral selection |
| *CcCPK7-CcCPK28* | 0.0000 | 0.0000 | 1.0000 | Neutral selection |
| *CcCPK11-CcCPK30* | 0.0633 | 0.6434 | 0.0984 | Purifying selection |

Ka: non-synonymous substitution rate; Ks: synonymous substitution rate.

**Table S5.** The Ka/Ks ratios for duplicated *CPK* genes in *C. nankingense*.

| **Paralogues** | **Ka** | **Ks** | **Ka/Ks** | **Selective Pressure** |
| --- | --- | --- | --- | --- |
| *CnCPK1-CnCPK18* | 0.0877 | 0.8383 | 0.1046 | Purifying selection |
| *CnCPK1-CnCPK26* | 0.0861 | 1.2007 | 0.0717 | Purifying selection |
| *CnCPK2-CnCPK30* | 0.0766 | 1.0030 | 0.0764 | Purifying selection |
| *CnCPK4-CnCPK16* | 0.0592 | 0.8445 | 0.0701 | Purifying selection |
| *CnCPK4-CnCPK17* | 0.0805 | 1.0429 | 0.0772 | Purifying selection |
| *CnCPK5-CnCPK28* | 0.2284 | 0.1682 | 1.3579 | Positive selection |
| *CnCPK14-CnCPK25* | 0.0047 | 0.0178 | 0.2640 | Purifying selection |
| *CnCPK16-CnCPK17* | 0.0790 | 0.7842 | 0.1007 | Purifying selection |
| *CnCPK16-CnCPK31* | 0.0275 | 0.0181 | 1.5193 | Positive selection |
| *CnCPK18-CnCPK26* | 0.2287 | 0.1409 | 1.6231 | Positive selection |
| *CnCPK20-CnCPK24* | 0.0828 | 0.1037 | 0.7985 | Purifying selection |
| *CnCPK29-CnCPK34* | 0.0071 | 0.0473 | 0.1501 | Purifying selection |
| *CnCPK32-CnCPK7* | 0.0051 | 0.0260 | 0.1962 | Purifying selection |

Ka: non-synonymous substitution rate; Ks: synonymous substitution rate.

**Table S6.** Tajima relative rate tests of CPK gene pairs in *H. annuus*^a^.

| **Testing Group** | **Group** | **Mt^b^** | **M1^c^** | **M2^d^** | **χ2** | ***p*^e^** |
| --- | --- | --- | --- | --- | --- | --- |
| HaCPK5/HaCPK30 with SlCPK26 | III | 427 | 7 | 15 | 2.91 | 0.08808 |
| HaCPK10/HaCPK25 with SlCPK12 | I | 455 | 14 | 11 | 0.36 | 0.54851 |
| HaCPK13/HaCPK32 with SlCPK20 | II | 401 | 8 | 12 | 0.80 | 0.37109 |
| HaCPK9/HaCPK19 with SlCPK28 | IV | 425 | 13 | 10 | 0.39 | 0.53161 |
| HaCPK12/HaCPK35 with SlCPK16 | II | 396 | 20 | 13 | 1.48 | 0.22302 |
| HaCPK26/HaCPK31 with SlCPK17 | II | 351 | 23 | 24 | 0.02 | 0.88403 |
| HaCPK33/HaCPK34 with SlCPK3 | I | 406 | 21 | 9 | 4.80 | 0.02846 |
| HaCPK21/HaCPK30 with SlCPK26 | III | 412 | 21 | 21 | 0.00 | 1.00000 |
| HaCPK36/HaCPK39 with SlCPK25 | III | 439 | 20 | 18 | 0.11 | 0.74560 |
| HaCPK2/HaCPK23 with SlCPK9 | I | 336 | 15 | 21 | 1.00 | 0.31731 |
| HaCPK5/HaCPK21 with SlCPK26 | III | 423 | 11 | 20 | 2.61 | 0.10600 |
| HaCPK18/HaCPK24 with SlCPK18 | II | 389 | 20 | 11 | 2.61 | 0.10600 |
| HaCPK1/HaCPK33 with SlCPK3 | I | 397 | 18 | 28 | 2.17 | 0.14037 |
| HaCPK5/HaCPK11 with SlCPK26 | III | 412 | 20 | 30 | 2.00 | 0.15730 |
| HaCPK11/HaCPK30 with SlCPK26 | III | 404 | 27 | 28 | 0.02 | 0.89274 |
| HaCPK33/HaCPK38 with SlCPK3 | I | 406 | 37 | 9 | 17.04 | 0.00004 |
| HaCPK9/HaCPK28 with SlCPK28 | IV | 401 | 31 | 18 | 3.45 | 0.06329 |
| HaCPK10/HaCPK16 with SlCPK12 | I | 418 | 24 | 46 | 6.91 | 0.00855 |
| HaCPK11/HaCPK21 with SlCPK26 | III | 407 | 26 | 25 | 0.02 | 0.88864 |
| HaCPK19/HaCPK28 with SlCPK28 | IV | 406 | 13 | 29 | 6.10 | 0.01355 |

^a^ The Tajima relative rate test was used to examine the equality of evolutionary rate between sunflower paralogues;

^b^ Mt is the sum of the identical sites in all three sequences tested;

^c^ M1 is the number of unique differences in the first paralogue;

^d^ M2 is the number of unique differences in the second paralogue;

^e^ If *p* < 0.05, the test rejects the equal substitution rates between the two duplicates and infers that one of the two duplicates has an accelerated evolutionary rate.

**Table S7.** Tajima relative rate tests of CPK gene pairs in *L. sativa*^a^.

| **Testing Group** | **Gruop** | **Mt^b^** | **M1^c^** | **M2^d^** | **χ2** | ***p*^e^** |
| --- | --- | --- | --- | --- | --- | --- |
| LsCPK28/LsCPK33 with SlCPK25 | III | 444 | 22 | 16 | 0.95 | 0.33039 |
| LsCPK19/LsCPK28 with SlCPK25 | III | 452 | 10 | 22 | 4.50 | 0.03389 |
| LsCPK19/LsCPK33 with SlCPK25 | III | 454 | 12 | 18 | 1.20 | 0.27332 |
| LsCPK1/LsCPK8 with SlCPK12 | I | 447 | 22 | 23 | 0.02 | 0.88150 |
| LsCPK20/LsCPK31 with SlCPK26 | III | 412 | 15 | 30 | 5.00 | 0.02535 |
| LsCPK20/LsCPK32 with SlCPK26 | III | 425 | 10 | 17 | 1.81 | 0.17793 |
| LsCPK21/LsCPK26 with SlCPK18 | II | 385 | 19 | 17 | 0.11 | 0.73888 |
| LsCPK31/LsCPK32 with SlCPK26 | III | 411 | 24 | 16 | 1.60 | 0.20590 |
| LsCPK11/LsCPK18 with SlCPK29 | IV | 410 | 47 | 17 | 14.06 | 0.00018 |
| LsCPK15/LsCPK29 with SlCPK8 | I | 444 | 30 | 14 | 5.82 | 0.01586 |
| LsCPK16/LsCPK18 with SlCPK29 | IV | 412 | 45 | 18 | 11.57 | 0.00067 |

^a^ The Tajima relative rate test was used to examine the equality of evolutionary rate between *Lactuca sativa* paralogues;

^b^ Mt is the sum of the identical sites in all three sequences tested;

^c^ M1 is the number of unique differences in the first paralogue;

^d^ M2 is the number of unique differences in the second paralogue;

^e^ If *p* < 0.05, the test rejects the equal substitution rates between the two duplicates and infers that one of the two duplicates has an accelerated evolutionary rate.

**Table S8.** Tajima relative rate tests of CPK gene pairs in *C. cardunculus*^a^.

| **Testing Group** | **Gruop** | **Mt^b^** | **M1^c^** | **M2^d^** | **χ2** | ***p*^e^** |
| --- | --- | --- | --- | --- | --- | --- |
| CcCPK6/CcCPK18 with SlCPK19 | I | 281 | 0 | 0 | 0.00 | 1.00000 |
| CcCPK7/CcCPK28 with SlCPK29 | IV | 425 | 25 | 15 | 2.50 | 0.11385 |
| CcCPK3/CcCPK15 with SlCPK25 | III | 450 | 20 | 14 | 1.06 | 0.30348 |
| CcCPK1/CcCPK27 with SlCPK26 | III | 429 | 9 | 15 | 1.50 | 0.22067 |
| CcCPK11/CcCPK30 with SlCPK8 | I | 450 | 19 | 13 | 1.13 | 0.28884 |
| CcCPK2/CcCPK25 with SlCPK18 | II | 390 | 19 | 20 | 0.03 | 0.87278 |
| CcCPK5/CcCPK23 with SlCPK20 | II | 388 | 29 | 32 | 0.15 | 0.70090 |

^a^ The Tajima relative rate test was used to examine the equality of evolutionary rate between *Cynara cardunculus* paralogues;

^b^ Mt is the sum of the identical sites in all three sequences tested;

^c^ M1 is the number of unique differences in the first paralogue;

^d^ M2 is the number of unique differences in the second paralogue;

^e^ If *p* < 0.05, the test rejects the equal substitution rates between the two duplicates and infers that one of the two duplicates has an accelerated evolutionary rate.

**Table S9.** Tajima relative rate tests of CPK gene pairs in *C. nankingense*^a^.

| **Testing Group** | **Group** | **Mt^b^** | **M1^c^** | **M2^d^** | **χ2** | ***p*^e^** |
| --- | --- | --- | --- | --- | --- | --- |
| CnCPK7/CnCPK32 with SlCPK28 | IV | 175 | 0 | 1 | 1.00 | 0.31731 |
| CnCPK14/CnCPK25 with SlCPK7 | I | 313 | 39 | 3 | 30.86 | 0.00001 |
| CnCPK29/CnCPK34 with SlCPK12 | I | 385 | 36 | 1 | 33.11 | 0.00001 |
| CnCPK16/CnCPK31 with SlCPK26 | III | 330 | 2 | 29 | 23.52 | 0.00001 |
| CnCPK16/CnCPK17 with SlCPK26 | III | 376 | 20 | 23 | 0.21 | 0.64731 |
| CnCPK20/CnCPK24 with SlCPK16 | II | 348 | 1 | 7 | 4.50 | 0.03389 |
| CnCPK1/CnCPK18 with SlCPK8 | I | 427 | 20 | 26 | 0.78 | 0.37634 |
| CnCPK2/CnCPK30 with SlCPK25 | III | 381 | 14 | 41 | 13.25 | 0.00027 |
| CnCPK4/CnCPK16 with SlCPK26 | III | 371 | 22 | 19 | 0.22 | 0.63941 |
| CnCPK4/CnCPK17 with SlCPK26 | III | 400 | 25 | 25 | 0.00 | 1.00000 |
| CnCPK1/CnCPK26 with SlCPK8 | I | 438 | 28 | 15 | 3.93 | 0.04743 |
| CnCPK5/CnCPK28 with SlCPK29 | IV | 413 | 20 | 29 | 1.65 | 0.19854 |
| CnCPK18/CnCPK26 with SlCPK8 | I | 429 | 36 | 18 | 6.00 | 0.01431 |

^a^ The Tajima relative rate test was used to examine the equality of evolutionary rate between *Chrysanthemum nankingense* paralogues;

^b^ Mt is the sum of the identical sites in all three sequences tested;

^c^ M1 is the number of unique differences in the first paralogue;

^d^ M2 is the number of unique differences in the second paralogue;

^e^ If *p* < 0.05, the test rejects the equal substitution rates between the two duplicates and infers that one of the two duplicates has an accelerated evolutionary rate.
